# Evaluation of Mechanical Properties of Glass Ionomer Cements Reinforced with Synthesized Diopside Produced via Sol–Gel Method

**DOI:** 10.3390/ma16052107

**Published:** 2023-03-05

**Authors:** Ali Maleki Nojehdehi, Farina Moghaddam, Bejan Hamawandi

**Affiliations:** 1 Department of Material Science, Tabriz University, Tabriz 5166616471, Iran; 2 Department of Applied Physics, KTH Royal Institute of Technology, SE-106 91 Stockholm, Sweden

**Keywords:** glass ionomer cement, diopside nanoparticles, mechanical properties, fluoride release

## Abstract

This study aimed to fabricate a glass ionomer cement/diopside (GIC/DIO) nanocomposite to improve its mechanical properties for biomaterials applications. For this purpose, diopside was synthesized using a sol–gel method. Then, for preparing the nanocomposite, 2, 4, and 6 wt% diopside were added to a glass ionomer cement (GIC). Subsequently, X-ray diffraction (XRD), differential thermal analysis (DTA), scanning electron microscopy (SEM), and Fourier transform infrared spectrophotometry (FTIR) analyses were used to characterize the synthesized diopside. Furthermore, the compressive strength, microhardness, and fracture toughness of the fabricated nanocomposite were evaluated, and a fluoride-releasing test in artificial saliva was also applied. The highest concurrent enhancements of compressive strength (1155.7 MPa), microhardness (148 HV), and fracture toughness (5.189 MPa·m^1/2^) were observed for the glass ionomer cement (GIC) with 4 wt% diopside nanocomposite. In addition, the results of the fluoride-releasing test showed that the amount of released fluoride from the prepared nanocomposite was slightly lower than the glass ionomer cement (GIC). Overall, the improvement in mechanical properties and optimal fluoride release of prepared nanocomposites can introduce suitable options for dental restorations under load and orthopedic implants.

## 1. Introduction

Glass ionomer cement (GIC) was invented in 1969 by Wilson and Kent in England in a chemical laboratory [1,2]. Since its invention, this cement has been used in clinical dentistry as a restorative biomaterial [3,4,5,6,7]. Glass ionomer cements (GICs) are organic base materials and are known as polyalkenoate cement. This work about these materials is based on the acid–base reaction between calcium fluoroaluminosilicate glass powder and an aqueous solution of polyacrylic acid [1,8]. The initial design of glass ionomer cements (GICs) was a formula of silicate and polycarboxylate cement. Glass ionomer cements (GICs) used aluminosilicate powder from silicate cement and polyacrylic acid liquid from polycarboxylate cement to have the properties of both types of cement together. Hardening of glass ionomer cement (GIC) takes place during three stages: dissolution, gelation, and hardening. Therefore, the hardening mechanism of glass ionomer cement (GIC) includes the dissolution of the surface of glass particles in polymer liquid, then the release of aluminum and calcium ions and finally, the formation of calcium and aluminum polyacrylate chains in the matrix of hardened cement [9].

Glass ionomer cement (GIC) has been used among restorative materials due to some desirable physical, chemical, and biological properties [10]. These materials have permanent adhesion to tooth enamel and dentin, they have the property of releasing fluoride for a long time, and when they are exposed to a solution containing fluoride, they are able to absorb and store it; therefore, they have anti-caries properties [1]. Good biocompatibility in the mouth, color matching with dentin and tooth enamel, acid resistance, thermal expansion coefficient similar to the tooth structure, non-shrinkage due to self-adherence, and non-toxicity are among the other characteristics of these cement [1,11]. Glass ionomers are generally widely used to restore dental structures in dentistry. Apart from dentistry, glass ionomer cement (GIC) has also been suggested as a material for use in bone repair surgeries [12]. In addition, D’Orto et al. [13] reported that replacing dental implants with new cement supporting fixed prostheses can also be a useful solution in patients with type I diabetes, provided compensation is performed and recent blood tests are checked by the clinician prior to surgery. Additionally, this replacement can be an effective treatment to prevent mucositis caused by radiotherapy and/or chemotherapy interventions at high risk of oral and pharyngeal mucosa damage [14]. Furthermore, to the advantages of glass ionomer cement (GIC), the most important limitation in the use of these cements is their weak mechanical properties, which limits their use in applications under high stress. Poor mechanical properties cause primary and secondary implant failure [15], which can be achieved by introducing new cement.

In recent years, many efforts have been made to improve the mechanical properties of glass ionomer cement (GIC) [16]. Many researchers have worked in this field with different ideas. These research include adding zirconia particles, bioglass, hydroxyapatite, fluor apatite, forsterite, and titanium diopside to glass ionomer cement (GIC) [1]. In a previous study, we reinforced glass ionomer cement by the forsterite, which showed good mechanical results [10] and was the beam of light for our next studies. Diopside (DIO), with the chemical composition CaMgSi_2_O_6_, is one of the important biomaterials that belongs to the group of pyroxenes. All three elements of silicon, calcium, and magnesium, which are needed in the development of the skeletal system, are found in the chemical composition of diopside (DIO). Due to its biocompatibility and mechanical properties, it is used in a wide variety of clinical applications, such as bone and dental root implants, surgery hemostasis applications, drug delivery, and in vivo imaging [17]. In addition, according to Nonami et al.’s research, the comparison of the mechanical properties and biocompatibility of diopside and hydroxyapatite proved that diopside (DIO) has better mechanical properties than hydroxyapatite. On the other hand, due to its good biocompatibility, it can be used in cases where the use of hydroxyapatite is limited [18]. According to the report of Khandan et al., even the produced hydroxyapatite-diopside (DIO) bio-nanocomposite coatings show favorable biocompatibility and high hardness [19]. Notwithstanding, diopside (DIO) nanoparticles are well known due to their mechanical properties, and they can create good wetting with water because of their superficial hydroxyl groups on their surface; therefore, they have been chosen as a substrate for adsorbents [20].

Another feature of diopside (DIO) is the possibility of obtaining these nanoparticles through natural waste sources such as rice husk as a source of silica and eggshell as a source of calcium oxide by the sol–gel method that is reported by Choudhary et al. [21]. The purpose of this study is to fabricate and characterize the diopside (DIO) glass ionomer cement (GIC) nanocomposite and compare the effect of adding these nanoparticles to the ceramic component of glass ionomer cement (GIC) to improve its mechanical properties. Expanding the use of these cement in dentistry and orthopedics due to the improvement of mechanical properties is one of the important achievements of this research. For this purpose, diopside (DIO) nanoparticles were prepared by the sol–gel method, and different weight percentages were added to the commercial glass ionomer cement (GIC) ceramic, and after mixing with cement polymer liquid, the manufactured nanocomposites were subjected to mechanical tests and fluoride release.

## 2. Materials and Methods

### 2.1. Synthesis of Diopside Nanoparticles

Magnesium nitrate (6Mg(NO_3_)_2_.6H_2_O), calcium nitrate (Ca(NO_3_)_2_.4H_2_O), tetraethyl orthosilicate (TEOS, SiC_8_H_20_O_4_), and ethanol in 99% purity were purchased from Merck, Germany. They were used as raw materials for the synthesis of diopside (DIO, CaMgSi_2_O_6_) by sol–gel method. To prepare diopside (DIO), the 29.52 g calcium nitrate and 32.05 g magnesium nitrate were added in the 92.14 g ethanol and stirred in a magnetic stirrer for 30 min at 80 °C. After dissolving the salts in ethanol, 52.08 g tetraethyl orthosilicate (TEOS) was added to the solution and stirred under slower rate at 30 °C for 30 min. The produced sol was placed in a dryer at 120 °C for 48 h. Then, the very agglomerated powder from the dryer was manually ground. Based on differential thermal analysis (DTA) results to achieve the desired nanopowder, the sol was heated with the rate of 10 °C/min at 800 °C for 2 h [22]. Then, we ground it by ball-mill. Finally, we reached diopside nanoparticle with 100 nm.

### 2.2. Manufacture of Nanocomposite Samples

Fuji II GC commercial glass ionomer cement (GIC) (a product of Corporation GC, Tokyo, Japan) was purchased. To prepare the nanocomposites, 2, 4, and, 6 wt% diopside nanoparticles were added to glass ionomer cement (GIC)’s powder portion and then mixed with polymeric liquid. First, glass ionomer cement powder was mixed with different weight percentages of diopside nanoparticles for 30 s in an amalgamator. Then, the mixed powders were distributed on the glass plate. Then, they were mixed with the liquid (acrylic acid) in a 2:1 ratio; the mixing method was followed according to the factory instructions under the recommended conditions and time. Through this way, at first, half of the distributed powder, using a plastic spatula, entered the polymer liquid slowly and was quickly mixed within 10 s. Then, the second part of the glass ionomer powder was completely added to the mixture, and this mixing took place within 15 to 20 s. After setting cement for 30 s [23], they were cast in cylindrical molds (6 mm diameter and 12 mm height) at room temperature. The final mixed material had a shiny and wet surface. In the end, the obtained dough was transferred to an aluminum mold containing grooves with specific dimensions according to the relevant standards. In this step, we tried to condense the resulting dough from one side of the mold wall to prevent the formation of air bubbles in the mixture. After the samples were completely set, they were removed from the molds and ground (P600 to P2000) to obtain smoother surfaces. Finally, they were used for tests.

### 2.3. Mechanical Tests

Mechanical tests were evaluated 24 h after setting cement. The whole assembly was stored at 36 °C and at least 95% relative humidity [24]. To perform the compressive strength test, cylindrical samples with a diameter of 6 ± 0.1 mm and a height of 12 ± 1.0 mm were prepared in accordance with the ISO 9917-1 standard. The force was applied to the sample along the longitudinal axis at a speed of 0.5 mm/min. The compressive strength was determined based on Equation (1) [25]:

(1) $$C=4P/{\pi d}^{2}$$

where C is the compressive strength (MPa), P is the maximum fracture force (N), and d is the sample diameter (mm). The Rockwell method is a rapid method for determining hardness of dental materials. Rockwell hardness number (RHN) is designated according to the particular indenter and load applied [26]. In this work, the microhardness test was done by a Rockwell C device by applying a 20 N load. Additionally, to determine the fracture toughness, KOOPA UV1 model macro hardness tester was used [10]. Through this method, we created a crack on the surface of the glass ionomer cement (GIC) 4 wt% diopside (DIO) nanocomposite because, at forces lower than this value, cracks do not form on the surface of the nanocomposite. Our goal was to calculate the toughness by creating a crack on the nanocomposite surface, with the relationship between the applied force and the crack length. It should be noted that the evaluation of the cracks by using an optical microscope and ImageJ software (v 1.53), and the value of the fracture toughness was calculated with the following relationship in this article. Therefore, cylindrical specimens with dimensions of 6 mm φ × 12 mm were mounted by polyester, and hardness effect on the surface was measured. Based on the crack length and applied force, the fracture toughness was calculated (Equation (2)) [27]. For each test, 5 samples were tested, and the average of data with error bar was reported.

K_IC_ = 0.0889 [Hv·P/∑c_i_]^1/2^ (2)

where K_IC_ is the fracture toughness, HV is microhardness, P is applied load, and C is crack length plus half diameter of the hardening effect.

### 2.4. Fluoride Release Assessment Test (ICP)

The fluoride release test was performed over a period of 14 days. The samples were prepared in the form of a cylinder with a diameter of 6 mm and a height of 12 mm. First, each sample was placed separately in a test tube containing 15 mL of artificial saliva. Plastic tubes were used in 15 mL packages because glass containers absorb and release fluoride. The chemical composition of artificial saliva used is given in Table 1. All materials release their highest proportion of cumulative total fluoride in the first 24 h after mixing [28]. The amount of fluoride released after the first, third, seventh, and fourteenth days of mixing was measured and recorded. At the end of each of these periods, after leaving the test tube, each sample was washed twice with deionized water. Then, to avoid saturation of the solution by fluoride ion after drying, the sample was placed in fresh artificial saliva solution. During the test period, the samples were kept in a bain-marie bath with a temperature of 37 °C [10]. After removing the test tubes, the fluoride sample of the artificial saliva solution was measured using a potentiometric method by a potentiometer (pH/ISE, Meter Thermo Orion, Waltham, MA, USA) and using a specific fluoride ion electrode (Fluoride Combination Electrode, 96-09-00, Thermo Fisher Scientific, Waltham, MA, USA). Before starting the measurement in each period, the potentiometer device was calibrated by fluoride standard solutions containing 0.1, 1, and 10 mg/L of fluoride ion, respectively. The calibration slope of the device was between 58.7 and 60.2. Before measurement, 1 mL of each solution was mixed with 0.1 mL of TISAB III buffer solution (Thermo Orion, Waltham, MA, USA) in a 5 mL polyethylene vial under ambient conditions using a magnetic stirrer [29]. A buffer was added to each solution to remove disturbing ions in the measurement of fluoride ion. To measure, first, the electrode was placed inside the solution, and the container containing the solution was shaken to make the diffusion of fluoride ion in the solution uniform. In the end, the concentration of fluoride ion in terms of ppm for the solution was read and recorded directly from the device. After this operation, the electrode was removed from the solution and completely washed with distilled water, and, after drying, it was used again to measure the next sample. The fluoride concentration of each solution was measured three times and on different days.

### 2.5. Characterizations

To analyze the phase composition and determine the grain size of the synthesized diopside powders, X-ray diffraction (Philips expert) was used. The X-ray diffraction was operated with voltage and current settings of 30 kV and 20 mA, and used Cu-Kα radiation λ = 1.5404 Å in the 2θ range from 10 to 80°. The grain size of the milled diopside powders was measured by evaluating the XRD peaks using Scherer Equation (3) [31]:

(3) $$\beta =\frac{k\lambda }{L\;\cos\theta }$$

β is the full width of the diffraction peak under consideration (rad.) at half maximum intensity, k is a constant (k = 0.89), λ (nm) is the wavelength of the X-ray, θ(°) is the Bragg diffraction angle, and (L) is the size of the grain (nm).

High magnification FESEM images (TESCAN is located in Brno, Czech Republic) was also used to study the particle size and morphology of the synthesized diopside powders. Additionally, Fourier transform infrared spectrophotometry (FTIR, Bruker Tensor 27 FTIR, Hardtstraße, Karlsruhe, Germany) analysis was performed to evaluate the amount and type of bonds of the material in question to ensure the desired synthesis result.

## 3. Results and Discussion

### 3.1. Characterization of Diopside (DIO) Nanoparticles

**Figure 1:**
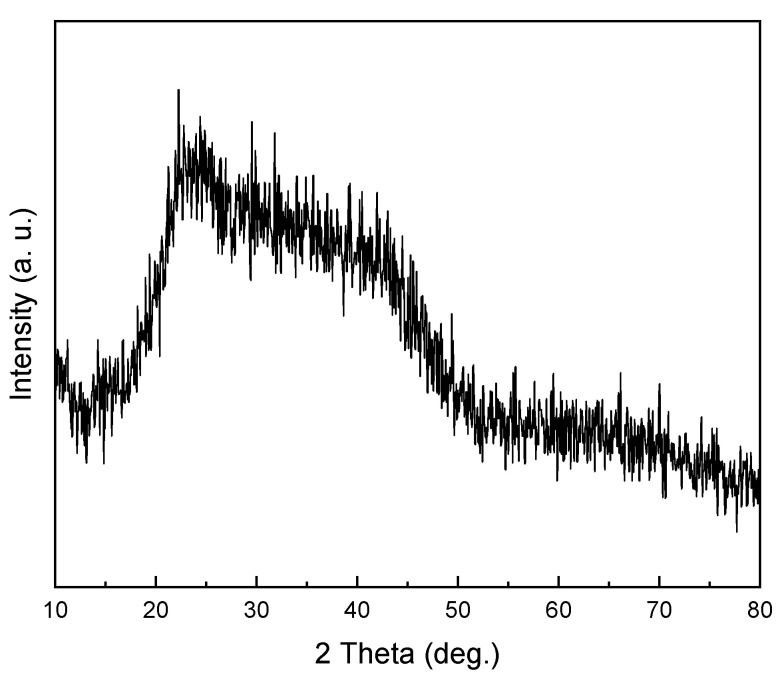
XRD pattern of dried diopside (DIO) powder at 120 °C for 24 h.

**Figure 2:**
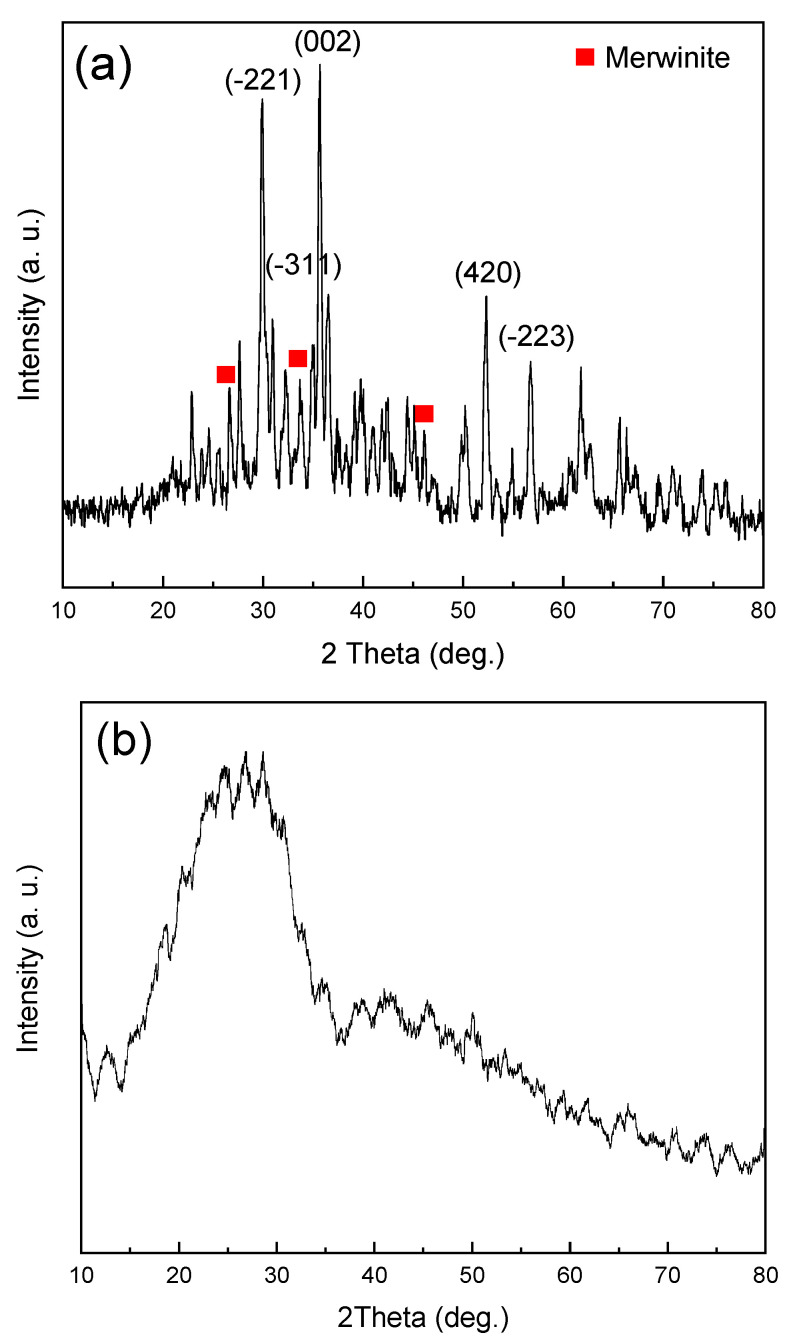
XRD pattern of (**a**) diopside (DIO) nanoparticles after calcination at 800 °C and (**b**) glass ionomer powder.

The XRD pattern of dried diopside (DIO) at 120 °C for 24 h is shown in Figure 1. As can be seen, the structure is amorphous, and no crystalline phase is created at this temperature. The XRD pattern of nanoparticles prepared by the sol–gel method, after calcination at 800 °C, is presented in Figure 2. The formation of the pure and completely crystalline phase of the diopside (DIO) particles was confirmed by comparing the angles and intensities of scattered peaks with the information contained in the standard JCDPS. Examining the available peaks shows that main peak at 29.9°, 35.5°, 56.7°, 54.9, and 30.8° correspond to the plans of (−221), (002), (−223), (420), and (−311), respectively, and are 100% compatible with the main peaks of the default diopside (DIO) in the 00-003-0860 card [29,32]. According to the phase analysis, there is ~97% diopside (DIO) and approximately 3% impurity. In previous studies, it has been proven that biomaterials based on calcium magnesium silicates, including diopside (CaMgSi_2_O_6_), akermanite (Ca_2_MgSi_2_O_7_), and merwinite (Ca_3_MgSi_2_O_8_), have good properties, such as high biocompatibility and biodegradability and superior mechanical characteristics [33]. Therefore, it was found that the presence of 3% of the merwinite (Ca_3_MgSi_2_O_8_) did not have a negative effect on the mechanical properties. The crystal size of the diopside (DIO) nanoparticles was calculated ~40 nm based on the modified Scherer equation [34]. The results obtained from the phase studies of the produced particles well show that the product of the sol–gel process and the subsequent heat treatment is nanocrystalline diopside (DIO) [35].

**Figure 3:**
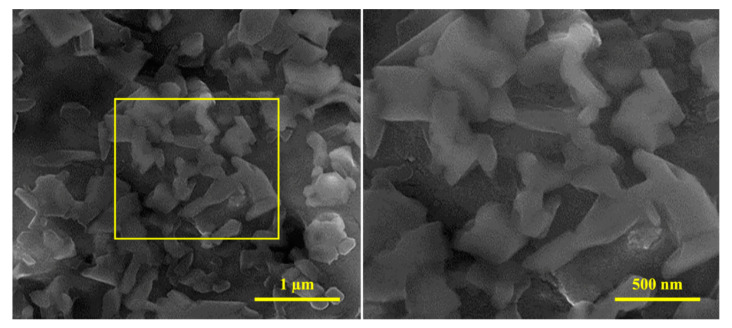
FESEM micrographs showing the size of the diopside (DIO) particles magnification at different magnification.

The FESEM micrographs of the dispersion of the diopside (DIO) particles are shown in Figure 3. The figure shows that the nanoparticles are formed in very small dimensions with no specific morphology. Similar to many nanometer particles, agglomerations are quite evident in FESEM micrographs due to high surface energy [36]. Achieving this morphology and particle size for diopside (DIO) particles caused by sol–gel method is consistent with the results of other researchers [37]. According to the different shapes and magnifications, the 200 particle sizes were measured in different areas, and it was observed that the particle size is about 140–150 nm, which is consistent with the crystallite size (~40 nm) calculated by XRD pattern. 

### 3.2. DTA of Synthesis Diopside (DIO)

**Figure 4:**
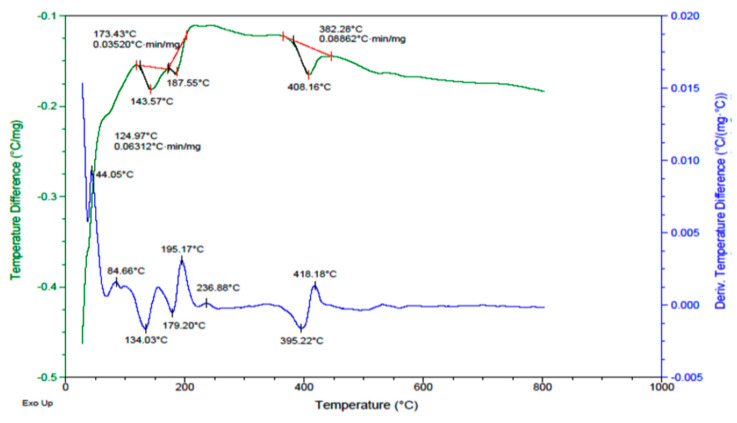
DTA-TGA of diopside particles dried at 120 °C.

The diopside (DIO) solution was dried at 120 °C for 24 h, and DTA was performed to determine the calcination temperature and the crystallization reactions. According to Figure 4, we can see an exothermic peak at ~550 °C, which indicates the heat reaction. Because the system tends to transform into a crystalline structure and lower its energy level. Consequently, it corresponds to our purpose and determines the calcination temperature.

### 3.3. FTIR Analysis of Diopside Nanoparticles

**Figure 5:**
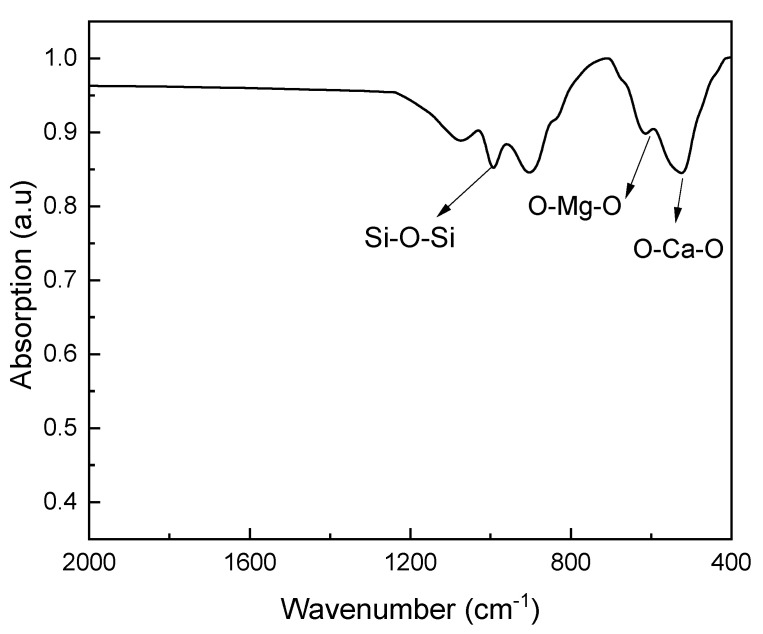
FTIR spectrum of diopside (DIO) nanoparticles.

Figure 5 shows FTIR spectrum of diopside nanoparticles. As shown in the figure, the peaks observed at 1000–1200 cm^−1^, 800 cm^−1^ and 600 cm^−1^ wavelengths correspond to the asymmetric tensile vibrations of the groups (Si-O-Si). Additionally, the peak corresponds to symmetric tensile vibrations (Si-O-Si) close to the wavelength of 800 cm^−1^ [25,38,39,40].

### 3.4. Evaluation of Fluoride Release

**Figure 6:**
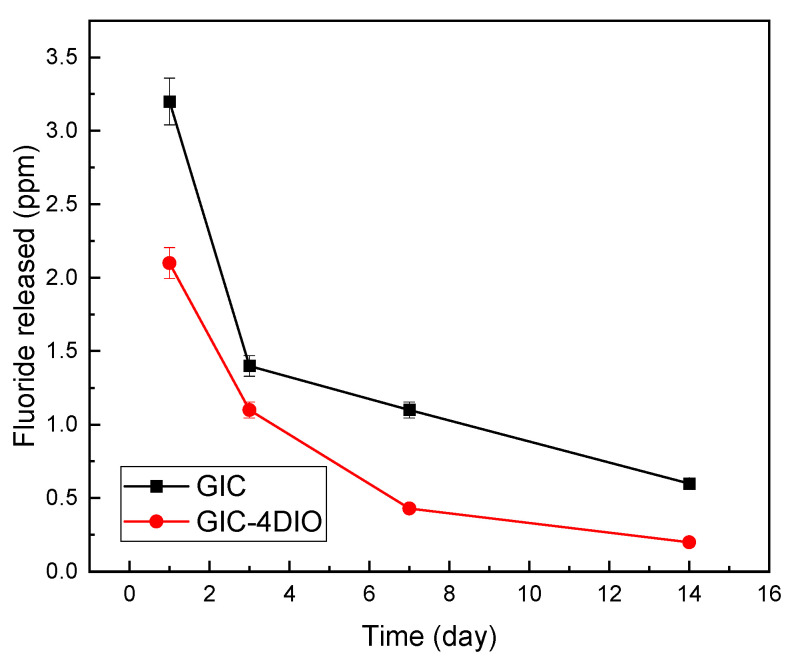
Amount of fluoride released from glass ionomer cement (GIC) and glass ionomer cement (GIC)-4 diopside (DIO) nanocomposite for 14 days immersion in artificial saliva.

The results of the fluoride release test by the ICP method, after 14 days of immersion of glass ionomer cement (GIC) and nanocomposite samples in artificial saliva solution are given in Figure 6. Figure 6 shows the initial high fluoride release on the first day and the slow and steady release of fluoride from the nanocomposite over time. The presence of diopside (DIO) nanoparticles in the glass ionomer structure do not interfere with the ability of glass ionomer cement (GIC) to release fluoride. Additionally, the graph shows that the amount of fluoride released for nanocomposites is less than pure glass ionomer cement (GIC) during the entire measurement period.

One of the factors influencing the release of fluoride from cement is the chemical composition of the ceramic component of glass ionomer cement (GIC). When glass powder is combined with diopside (DIO) nanoparticles and then mixed with polymer liquid, in addition to the surface dissolution of glass particles, the surface of diopside (DIO) nanoparticles is also dissolved in the presence of polymer liquid and calcium, magnesium and even silicon cations from the surface of diopside (DIO) nanoparticles is released. Calcium released from the surface of diopside (DIO) nanoparticles participates in the initial hardening mechanism of glass ionomer cement (GIC) and ultimately leads to the formation of more and stronger calcium and aluminum polyacrylates in the matrix of glass ionomer cement (GIC). In this case, the diffusion of fluoride from the glass ionomer cement (GIC) base is slow and requires more time. Therefore, the release of fluoride from cement decreases. It is important to note that the reactivity of sodium is higher than that of magnesium. Therefore, fluoride is mostly released in the form of sodium fluoride from glass ionomer cement (GIC). It seems that by immersing pure glass ionomer cement (GIC) in artificial saliva solution, more fluoride is available in the form of sodium fluoride, and thus the formation of fluoride is accelerated in the presence of sodium, and the tendency to release fluoride ions is reduced in the presence of diopside (DIO) nanoparticles.

**Figure 7:**
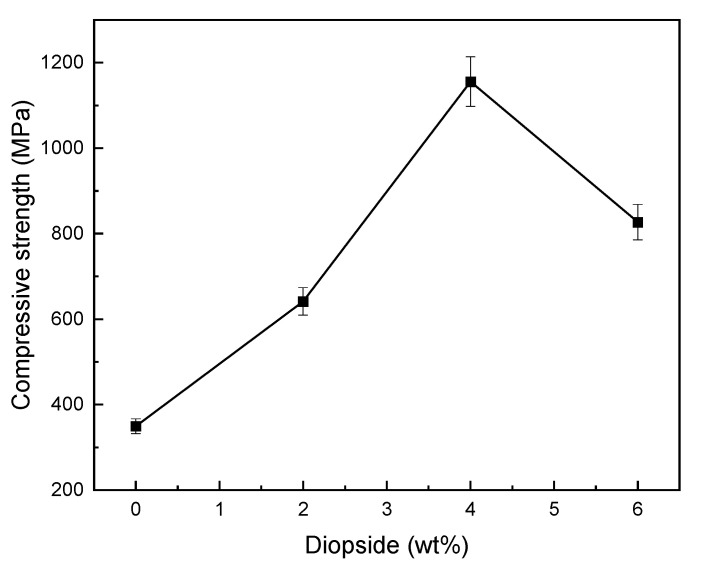
Compressive strength of glass ionomer and glass ionomer diopside with different weight percents.

Another factor affecting the release of fluoride from glass ionomer cement (GIC) is the porosity [41]. By adding diopside (DIO) nanoparticles to the ceramic component of glass ionomer cement (GIC), there will be a wider distribution of particle size in the structure of glass ionomer cement (GIC), which will result in greater density of powder particles mixed with the matrix cement polymer and increase mechanical properties of cement. Consequently, the diopside (DIO) particles will occupy the porosity between the glass cement particles and lead to a decrease in the porosity of the glass ionomer cement (GIC) structure [35,42,43]. By reducing the amount of porosity in the cement structure, the entry of artificial saliva into the cement is reduced, and as a result, less fluoride is released from the cement. Sumit et al. [44] investigated the relationship between the compressive strength of glass ionomer cement (GIC) and the amount of fluoride released from the cement. Their results showed that there is an inverse relationship between fluoride release and compressive strength, which is also consistent with the results presented in this research. According to Figure 3, Figure 4, Figure 5, Figure 6 and Figure 7, diopside (DIO) glass ionomer cement (GIC) nanocomposite has a higher compressive strength and lower fluoride release than pure glass ionomer cement (GIC).

### 3.5. Mechanical Properties

Figure 7 shows the compressive strength of glass ionomer cement (GIC) diopside (DIO) nanocomposites with different diopside (DIO) content. The results showed that by increasing the diopside (DIO) nanoparticles content up to 4 wt%, the compressive strength increases, but, at higher values (6 wt%), the compressive strength decreases. The glass ionomer cement (GIC) 4 wt% diopside (DIO) nanocomposite has the highest compressive strength compared to other samples, so the compressive strength of composite glass ionomer cement (GIC)-4 wt% diopside (DIO) compared to the plain sample increased from 349.5 MPa to 1155.7 MPa shows ~230% increase in compressive strength. In addition, by adding 2 wt% and 6 wt% of diopside (DIO) nanoparticles, the compressive strength increased by 42% and 98%, respectively, which shows adding diopside (DIO) nanoparticles in quantities less/more than 4wt% glass ionomer cement (GIC) will not have a destructive and reducing effect on the compressive strength of the cement, and it will be higher than the compressive strength of the plain sample. By comparing the results for glass ionomer cement (GIC) 4 wt% diopside (DIO) nanocomposite with the results reported for glass ionomer cement (GIC) 2 wt% forsterite nanocomposite [10], it is clear that the compressive strength has increased by 36%.

**Figure 8:**
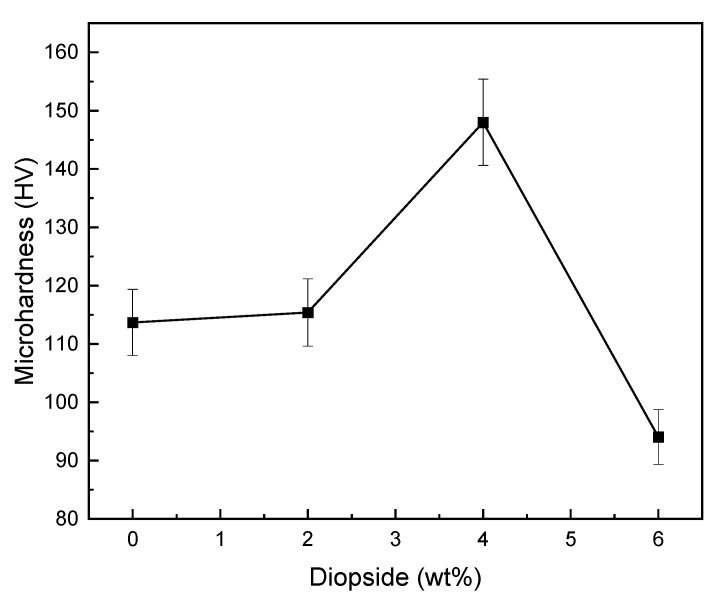
Microhardness of glass ionomer and glass ionomer diopside nanocomposites with different weight percentages.

Figure 8 illustrates the microhardness of glass ionomer cement (GIC) diopside (DIO) nanocomposites with different diopside (DIO) content. The results showed that the glass ionomer cement (GIC) 4 wt% diopside (DIO) nanocomposite has the highest microhardness compared to other nanocomposite samples, and compared to the plain cement, the microhardness increased from 113.7 HV to 148 HV, which means ~30% increase in microhardness. Moreover, the addition of diopside (DIO) nanoparticles less than 4 wt% glass ionomer cement (GIC) will not have a destructive and reductive effect on the microhardness, and the microhardness has increased by ~53%. By adding more than 4 wt% of diopside (DIO) nanoparticles to glass ionomer cement (GIC), the microhardness is lower than the initial microhardness value. By comparing the results for glass ionomer cement (GIC) 2 wt% diopside nanocomposite with the results reported for glass ionomer cement (GIC) 2 wt% forsterite nanocomposite [10], it indicated that almost the same microhardness is obtained.

**Figure 9:**
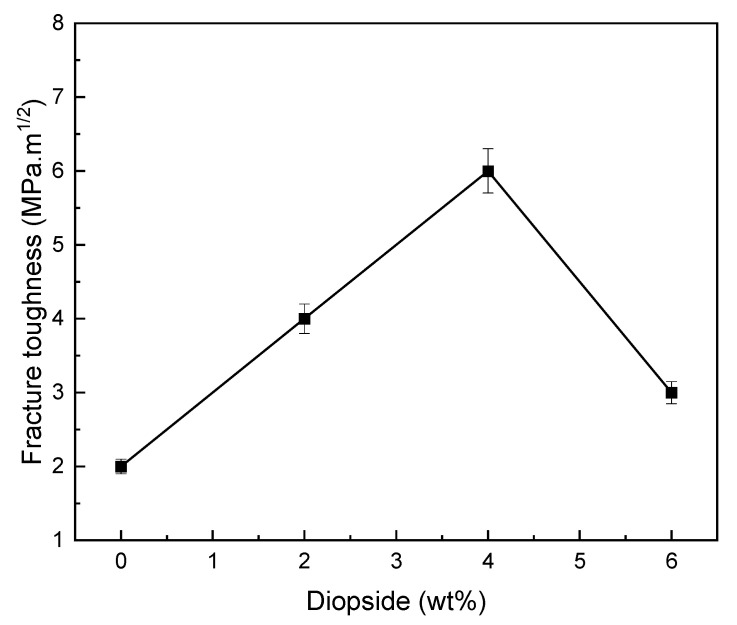
Fracture toughness of glass ionomer and glass ionomer diopside with different weight percent.

**Figure 10:**
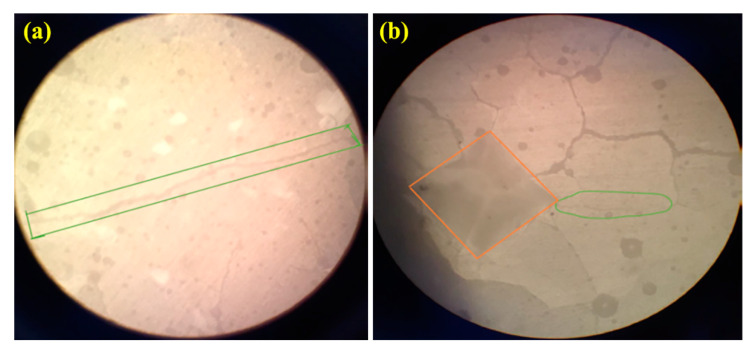
Optical microscopy images of the hardness effect under 200 N and the cracks created at the hardness outline of the: (**a**) glass ionomer cement (GIC), and (**b**) glass ionomer cement (GIC) 4 wt% diopside (DIO) composite.

Figure 9 and Figure 10 show fracture toughness of glass ionomer cement (GIC) diopside (DIO) nanocomposites with different diopside (DIO) content. The results showed that the glass ionomer cement (GIC) 4 wt% diopside (DIO) nanocomposite has the highest fracture toughness compared to other nanocomposite samples. The fracture toughness increased from 2.743 MPa.m^1/2^ to 5.189 MPa.m^1/2^, which means there is about an 89.17% increase in fracture toughness. Additionally, adding diopside (DIO) nanoparticles in quantities less than 4 wt% glass ionomer cement (GIC) did not have a destructive and reductive effect on the fracture toughness. By adding more than 4 wt% diopside (DIO) nanoparticles to the glass ionomer cement (GIC), the fracture toughness was not lower than the initial fracture toughness. Figure 7, Figure 8 and Figure 9 show that by adding the diopside (DIO) nanoparticles up to 4 wt%, the compressive strength, microhardness, and fracture toughness of glass ionomer cement (GIC) significantly increased.

In justifying the improvement of the mechanical properties of glass ionomer diopside cement nanocomposite in the presence of specific amounts of diopside nanoparticles, the following reason can be mentioned: Diopside particles have high mechanical properties. In most research that aim to improve the mechanical properties of the material, the addition of diopside as a secondary phase has been observed in the matrix. By adding diopside particles to the ceramic component of glass ionomer cement, these particles participate in the hardening mechanism of glass ionomer cement in such a way that in the acid and base reaction between the particles of Acrylic acid glass and polymer liquid, the surface of diopside particles dissolves due to the attack of acidic proton (polymeric liquid) H^+^, and Ca^2+^ ions are released from the surface of diopside particles. Therefore, more calcium ions will be available to form crosslinks and acrylate polysalts, which will strengthen the underlying matrix of glass ionomer cement and lead to an increase in cement strength [45,46]. Diopside nanoparticles prepared by the sol–gel method have a crystalline phase structure. Therefore, another reason for increasing the mechanical properties of glass ionomer cement in the presence of diopside nanoparticles is the formation of crystalline phases in the amorphous matrix of glass ionomer cement. By adding nanometer-sized diopside particles to glass ionomer cement powder with larger particle sizes than diopside particles, a wider distribution of particle size will occur in the structure of glass ionomer cement, which results in a greater density of powder particles mixed with the cement polymer matrix, and after that, it will bring better mechanical properties of cement. Diopside particles occupy the empty spaces between the cement glass particles and strengthen the cement base by creating more places to bond with carboxylic acid groups.

The loss of strength in nanocomposites containing diopside nanoparticles in amounts greater than 4 wt% for compressive strength, microhardness, and fracture toughness is due to the reduction of bonding and connecting forces between the ceramic and polymer components of glass ionomer cement. In fact, external diopside particles act as a barrier and prevent the complete connection of glass ionomer cement components. Diopside with nanometer particle size has more surface area compared to glass powder with micron particle size. In the presence of excessive amounts of diopside nanoparticles, the surface dissolution of these particles by polymer liquid is more than the surface dissolution of glass particles. This leads to the decrease in the formation of aluminum polyacrylates, which play a very important role in the final strength of glass ionomer cement. On the other hand, it is possible that the decrease in strength in the presence of excessive amounts of diopside nanoparticles indicates the lack of proper wetting in the interface of matrix and reinforcing particles because, with an increasing amount of nanoparticles in a cement matrix and with increasing surface area, the number of carboxylic acid groups available to bond with nanoparticles decreases. In this case, cracks are created around diopside nanoparticles and with the increase in diopside nanoparticles, the number of cracks in the interface of the matrix and the reinforcement increases. These cracks act as a stress concentration and lead to a decrease in mechanical properties [47,48,49,50]. The results of the mechanical test showed that by adding diopside nanoparticles to glass ionomer cement of up to 4 wt%, the compressive strength, microhardness, and fracture toughness of the nanocomposite produced is higher compared to glass ionomer cement; therefore, adding diopside nanoparticles to glass ionomer cement up to the above amounts is unimpeded.

### 3.6. FESEM Images and EDS Analysis of Glass Ionomer and Glass Ionomer 4 wt% Diopside Nanocomposites

**Figure 11:**
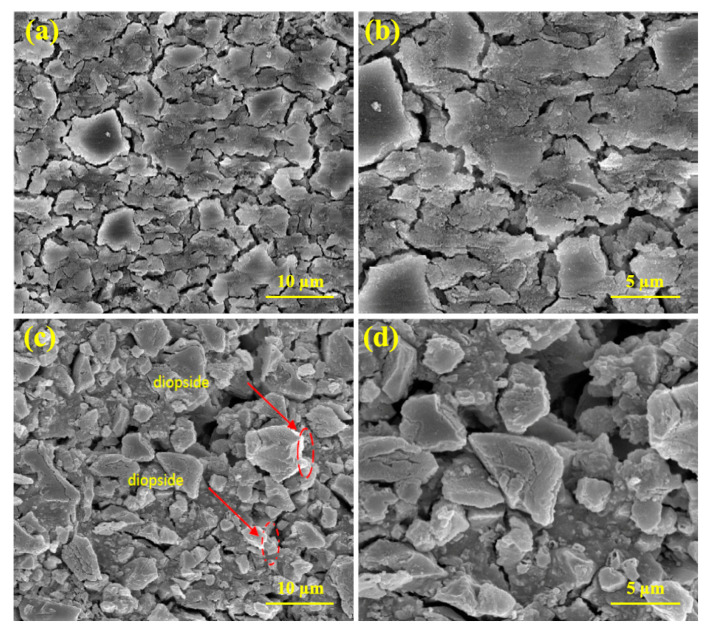
FESEM images of glass ionomer and glass ionomer nanocomposites with 4 wt% diopside: (**a**,**b**) Glass ionomer nanocomposite, and (**c**,**d**) Glass ionomer diopside nanocomposite at different magnifications.

According to the Figure 11, it can be seen that the microstructure of the glass ionomer nanocomposite without the reinforcing phase is in the form of a sheet with irregular corners. By adding diopside nanoparticles, these particles are placed between the particles of the matrix phase and bond with the surrounding particles, and they increase the mechanical properties of the nanocomposite. Additionally, the uniform distribution of the reinforcing phase is observed on the surface of the nanocomposite.

**Figure 12:**
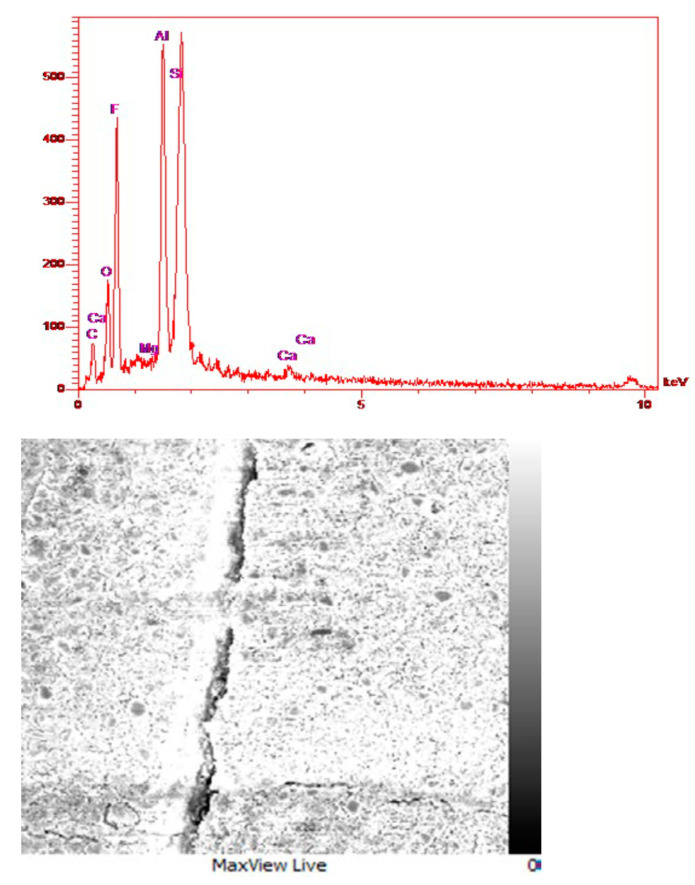
EDS and FESEM analysis of glass ionomer cement and distribution of elements on the nanocomposite surface.

**Figure 13:**
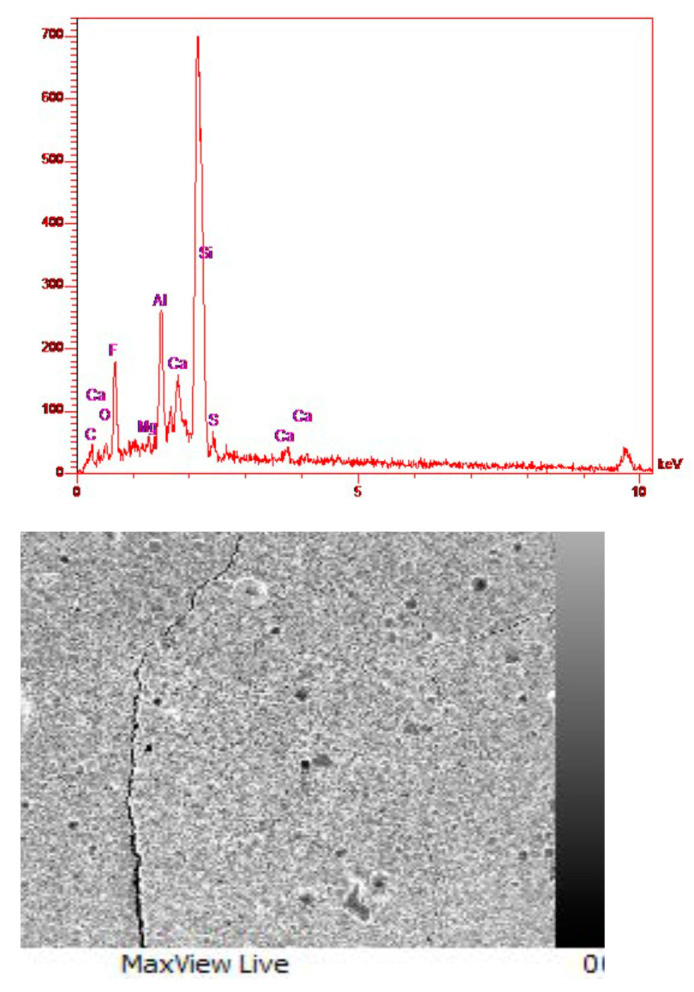
EDS and FESEM analysis of glass ionomer diopside nanocomposite and distribution of elements on the nanocomposite surface.

According to the components of glass ionomer cement, which are AlPO_4_, NaF, CaF_2_, AlF_3_, Al_2_O_3_, and SiO_2_, it can be seen that in the composition of glass ionomer cement, the peak of magnesium is very small and minor (Figure 12). Furthermore, according to the composition of diopside and forsterite that both have Mg, in the EDS analysis of glass ionomer cement and glass ionomer diopside nanocomposites, this peak has become a little more intense (Figure 13). Additionally, according to the structure of diopside, which contains calcium, the peak related to Ca is observed in the relevant analysis. In general, as expected in all three graphs, the highest peak is related to Si because the main composition of glass is SiO_2_ and diopside also contain Si. In order to justify the absence of a long peak for oxygen, which is one of the main elements of glass and diopside, it can be said that oxygen is relatively light in weight, and EDS and XRF analysis are not able to detect and identify it accurately.

The results of this study were also compared to previous studies on the effect of diopside, zirconium oxide, titanium oxide, and aluminum oxide nanoparticles on the properties of glass ionomer cement, as shown in Table 2. It is apparent that the addition of diopside nanoparticles has significantly improved the properties of glass ionomer.

## 4. Conclusions

In the current study, diopside (DIO) nanoparticles were synthesized, and subsequently, glass ionomer (GIC) diopside (DIO) cement nanocomposites were prepared, and the effect of adding diopside (DIO) nanoparticle on its mechanical properties and fluoride release was investigated. In comparison with previous studies, the obtained results from this research prove that the addition of diopside nanoparticles improves the properties of glass ionomer cement compared to other nanoparticles. The most important results of this study are as follows:

1. Phase analysis confirms that pure and crystalline diopside (DIO) nanoparticles were synthesized by the sol–gel method;
2. The optimal percentage of diopside (DIO) nanoparticles to increase the compressive strength, microhardness, and fracture toughness of glass ionomer cement (GIC) was 4 wt%, so the compressive strength, microhardness, and fracture toughness of glass ionomer cement (GIC) saw increases of about 230%, 30%, and 89%;
3. Adding diopside (DIO) nanoparticles to the ceramic component of glass ionomer cement (GIC) causes a slight decrease in the amount of fluoride releases;
4. The produced glass ionomer (GIC) diopside (DIO) cement nanocomposites, due to their mechanical properties, and favorable fluoride release, are suggested as a suitable option for dental restorations and orthopedic implants under load.

## Figures and Tables

**Table 1:** Synthetic saliva composition used in fluoride release test [30].

Components	Amount (mg/L)
NaCl	125.6
KCl	963.9
KSCN	189.2
KH_2_PO_4_	654.5
CaCl_2_.2H_2_O	227.8
NaHCO_3_	630.8
Na_2_SO_4_.10H_2_O	763.2
NH_4_Cl	178
Urea	200
pH	6.8

**Table 2:** Comparison of data with other researchers.

Group	Fracture Toughness (MPa m^1/2^)	Compressive Strength (MPa)	Surface Microhardness (VHN)	Fluoride Release	Ref.
GIC+3%TiO_2_	1.29	176.27	48.34	14.96 (μg/cm^2^)	[51]
GIC+5%Al_2_O_3_	_	190.57	96.23	_	[52]
GIC+3%ZrO_2_	_	_	88.8	_	[53]
GIC 5% nano ZrO_2_–SiO_2_–Hydroxyapatite	_	144.12	_	_	[54]
GIC/DIO	5.189	1155.7	148	2.1 (ppm)	This work

## Data Availability

Not applicable.
